# An Innovative Master in Anatomy: Combining Anatomy With Educational Scholarship

**DOI:** 10.1177/23821205231183866

**Published:** 2023-06-15

**Authors:** Alireza Jalali, Christopher J. Ramnanan

**Affiliations:** 1 Department of Innovation in Medical Education, Faculty of Medicine, 6363 University of Ottawa, Ottawa, ON, Canada

**Keywords:** anatomy, dissection, POCUS, medical education

## Abstract

The purpose of this article is to describe the design of a unique, bilingual (English and French) Master of Applied Sciences (M.Sc.) in Anatomical Sciences Education (ASE) program at a Canadian postsecondary institution. Anatomy is a core foundational discipline that is essential to many undergraduate, graduate, and professional programs in the health sciences. However, the number of new individuals with the necessary knowledge base and the pedagogical training to teach cadaveric anatomy are in short supply and cannot satisfy the number of openings for trained educators in the field. The M.Sc. in ASE was created to meet the increasingly critical need for instructors trained in human anatomy. The program is designed to prepare students for a career teaching human anatomy to students in the health sciences, emphasizing hands-on cadaveric dissection. Moreover, this program aims to develop educational scholarship skills in trainees by leveraging faculty expertise in medical education research, particularly in the field of anatomy education research. This focus on scholarships will make graduates more competitive for future faculty positions. During their first year of the program, learners will develop clinically relevant anatomy knowledge, teaching skills, and anatomy education scholarship. In the second year, students will benefit from an immediate, hands-on application of this acquired knowledge. In the same year, students will serve as anatomy teachers in the faculty's Medical Program and conduct their education scholarship projects, culminating in a formal research paper. Although similar programs have been developed in recent years, this article provides the first description of the creation of a graduate program in anatomy education. It includes needs assessment, program development, challenges faced, and lessons learned during the approval process. The article serves as a valuable resource for other institutions aspiring to develop similar initiatives.

## Introduction

Anatomy is a core foundation discipline for many undergraduate, graduate, and professional programs in the health sciences. Despite the ubiquitous nature of anatomy as a required course in university-level life science degrees, the number of people who are trained and prepared to teach anatomy at the postsecondary level has been precipitously declining globally.^1^ For at least 2 decades, new individuals qualified to teach anatomy (those with the necessary knowledge base and the pedagogical training to apply best teaching practices at an expert level as well as expertise in teaching anatomy using human cadaveric donors) have been in short supply and cannot fill the number of openings for trained educators in the field.^1^

To help address the need for individuals capable of teaching human anatomy, several graduate-level anatomical sciences education (ASE) programs have been developed in the last 2 decades, mainly in the United States.^2^ These programs have typically leveraged the local expertise of professors experienced in delivering human anatomy education to local health care programs (medical, dental, and allied health) and existing infrastructure (eg, cadaveric laboratories and body donation programs) to deliver anatomy instruction to graduate students in ASE. These graduate programs have evolved to complement cadaveric anatomy-related knowledge and teaching expertise with education in related disciplines such as embryology, neuroanatomy, and histology. This will be beneficial as many position descriptions state that some degree of teaching versatility is desirable in potential hires.^1,3^ In more recent years, an enhanced focus has been placed on developing educational research and scholarship skills in future anatomists because these skills are highly desired by hiring institutions.^3^

Although several ASE programs are already in place, most of them are situated in the United States, and scarce information has been published in terms of program evaluation regarding the successes and lessons learned from these initiatives. A program evaluation study was recently conducted on the 16-month Master of Science in Anatomical Sciences at Queen's University, aimed to prepare trainees as anatomy educators.^4^ Graduates from the first ten years of the program mentioned several strengths of their training, including the development of transferable skills such as pedagogical expertise, public speaking, and collaboration. However, graduates also noted several areas of potential improvement, including the fact that they would have benefitted from (i) a greater depth of education scholarship training, (ii) more opportunities to apply education scholarship training in formal dissemination environments, and (iii) increased training related to best teaching practices.^4^ Relatedly, the scholarly output of graduates in master-level ASE programs across North America remains poorly characterized. Finally, current training opportunities in ASE appear to be provided entirely in English.^2^ This is a critically important limitation in Canada given the need to develop anatomy educator competencies in French-speaking teachers to address anatomy-related course and program needs at French-speaking or bilingual colleges and universities.

With these considerations in mind, the purpose of this paper is to characterize the development of a novel Master of Applied Sciences in ASE program planned to begin in September 2023 in the University of Ottawa's Faculty of Medicine. This descriptive article will capture key elements during the program's conception, from the needs assessment stage to program approval, and considerations and lessons learned along the way. The resulting program will offer complete training in either French or English and emphasize knowledge application. Trainees will apply the anatomical knowledge they developed in their first year in the program in year 2 when they serve as anatomy educators in the faculty's Doctor of Medicine (MD) Program. Trainees will then apply their training in education scholarship to their research projects, culminating in a research paper at the end of their second year of study.

## Program development

### Needs assessment

In January 2019, a needs assessment was conducted involving one of the Faculty of Medicine's bachelor's degree programs, Transitional and Molecular Medicine (TMM). The TMM program was designed for upper-year undergraduate students interested in developing their applied skills in translational research and developing students who would be successful in graduate programs in the biomedical sciences. While this program initially aimed to develop strong biomedical researchers, the main career path for TMM alumni to date has been the healthcare professions (in particular, medical school), which mirrors a primary outcome of existing graduate programs in anatomy education. With these considerations in mind, the TMM students were queried regarding the need for a Master of Applied Sciences in Anatomical Science Education and what such a program should include and emphasize (Table 1). Twenty-four students participated in the survey. The survey used validated needs assessment survey items,^5^ adapted to this proposal, and invited participants to offer qualitative feedback. Item 1 demonstrated the perceived need for a program dedicated to the anatomical sciences. Participants surveyed expressed that a potential master-level program in ASE would address a gap in local education. Further, such a program would provide immense value to future healthcare professionals in terms of providing foundational knowledge necessary for clinical competence and the safe treatment of patients. Surveyed TMM students also stated that an anatomy-education training program should emphasize hands-on, cadaveric-dissection-based education and training related to evidence-based best practices in teaching. Finally, respondents generally expressed that select courses should be made available to graduate students registered in other faculties’ graduate programs.

**Table 1.** Summary of Findings From Needs Assessment Survey of Translational and Molecular Medicine Students (Conducted in January 2019).

ELEMENT	SUMMARY OF KEY FINDINGS (FROM LIKERT SCALE ITEMS AND OPEN-ENDED COMMENTARY)
Need for program	- Strong need for training in anatomical sciences - No comprehensive human anatomy program currently exists locally
Most likely beneficiaries of program	- Individuals aiming to develop knowledge base in anatomical sciences - Value to future health care professionals
Desired components that program should emphasize	- Hands-on, cadaveric-dissection-based training - Evidence-based pedagogical training - Courses made available to graduate students from other programs

### Program design, anatomical sciences component

Building from the needs assessment, this program was designed with an emphasis on hands-on dissection-based courses that mirror the sequence and content of prosection-based anatomy education in the faculty's MD Program. The suggested graduate program would mirror the MD program anatomy education sequence in chronology but not necessarily in content. Early consultations with anatomy faculty led to the realization that the graduate program (with different endpoints to consider) would have to have more comprehensive, detailed anatomy content, in certain areas compared to the MD Program. Head and neck and reproductive/pelvic anatomy were areas where the graduate program needed to emphasize, in far greater detail than what was being delivered to medical students. This organization of content delivery would allow for a comprehensive, full-body anatomy education experience in dissection in the first year of the program and then allow trainees to participate as laboratory teachers in all aspects of the MD Program in their second and final years. Early consultations among relevant Faculty and program organizers advocated for the inclusion of a broad array of anatomical science content, including histology, embryology, and pathology. Furthermore, because many job postings characterize an ideal candidate as being able to teach in related anatomical disciplines,^1^ year 1 course were also designed to provide training in histology, embryology, and pathology in environments that also emphasize hands-on, lab-based activities to provide learners with this foundational knowledge that would be required to successfully teach this content. Finally, a boot camp-like introductory course in point-of-care ultrasound technology in anatomy education was included given this tool's growing prominence in the healthcare professions and its relation to anatomy education.^6^ Early consultations also led to the consensus that content delivery (knowledge acquisition, from the student perspective) in these courses would need to be complemented with science communication and presentation opportunities and independent research components wherever possible. These elements would help support program-level learning outcomes relevant to developing educator- and/or education scholar-specific competencies. Overall, all courses in the anatomical sciences were created to emphasize student-centered learning approaches, which have been proven to stimulate engagement, participation, and teamwork in the MD Program.^7–9^ Instructors of individual courses would be encouraged to implement flipped classroom pedagogy as much as possible, which would have the added benefit of creating in-class opportunities for the application of knowledge in addition to increased time dedicated to hands-on activities, such as cadaveric dissection.^9,10^

### Program design, educational scholarship component

Much like comparable existing programs in anatomy education, the proposed program aimed to incorporate pedagogical training that would allow graduates to develop and apply best practices in their teaching activities. As alluded to above, even courses dedicated to knowledge acquisition and training in the anatomical sciences would incorporate presentation components (eg, journal club-style presentations) related to best practices in pedagogy related to the specific course. However, several existing master-level anatomy educator programs have been poorly characterized in terms of providing a substantive education research experience and this characterization may be an area of concern in existing programs.^4^ To address this concern, initial drafts of the program emphasized the importance of beginning training in education research as early as possible. At the start of the program (semester 1 of year 1), students will be registered in the immersive and established Introduction to Research in Education course offered through the Faculty of Education. This course will offer a broad, foundational introduction to education research that students can then build on throughout their studies. This course will also include an introduction to common principles and methodologies commonly used in medical education. Additionally, in Semester 5 of the program (during year 2), students will participate in the Faculty of Education's Seminar in Health Professions Education course, allowing for critical examination of applied research in a seminar format, as well as providing students with the opportunity to develop scientific communication skills by presenting a seminar focused on their research projects.

By the beginning of semester 2 of year 1, students will have chosen a research mentor and will begin their research projects, thereby permitting 4 complete semesters to be dedicated to their research paper. To assist with being matched with research mentors, available research projects and research aims of faculty will be collated and disseminated to students during the first semester. There are also networking events to help familiarize students with potential mentors during the program's first semester. To fit with the expertise within the home department, all projects will be focused on education science with faculty mentors who are experts in different fields of education. This extended research period was designed to address potential issues with research outputs derived from programs with shorter research durations4 and would therefore provide trainees with more complete, immersive research experiences. Ideally, at the end of their second year of study and completion of the program, students’ research projects will culminate in research papers and related scholarly products such as international presentations and/or peer-reviewed publications.

### Faculty recruitment

From the first iterations of this program, it was clear that it would have 2 points of emphasis: i) training in laboratory-based anatomical sciences and ii) pedagogical training and education research. The primary faculty for anatomical-sciences-related courses would be the established professors in the Division of Clinical and Functional Anatomy who have long contributed to the delivery of basic science education in the Faculty's MD program. On the other hand, students would receive pedagogical training by registering in previously established courses offered through the Faculty of Education and led by education professors. While not every Faculty of Education professor involved in course delivery may be an expert in medical education specifically, these professors would have expertise in the teaching of pedagogical principles and education research principles that are commonly employed in medical education. Regarding research mentorship, students would have a wide array of options for potential supervisors from the Faculty of Education and from Medicine's own Department of Innovation in Medical Education (in addition to anatomy educator scholars, this department has a growing list of professors with research interests that would likely overlap with those of students).

### Optimization during the external review process

During the External Review Process, 2 external reviewers (both bilingual, both with expertise in graduate-level education programs, and health professional education programs) reviewed program documents and were provided an online ‘site visit’ (as was called for, during the pandemic era approaches to travel and meetings) in April 2021 that included interviews with relevant students, faculty, program organizers, and administrators. While the external reviewers recognized the need and strengths of the program proposal, including the emphasis on bilingualism, and the immediate application of knowledge in both education scholarship and anatomical science expertise, they also articulated constructive suggestions in the following areas.

### Increasing content related to pedagogy and education

The external review process brought forward thoughtful and insightful suggestions to improve the program. In terms of program content, external reviewers agreed that trainees would benefit from additional training in pedagogy. These consultations resulted in the incorporation of established Faculty of Education courses that would provide benefits specific to future anatomy educators. The first of these courses (Technology and Health Professions Education) would provide training regarding best practices in utilizing modern teaching technologies in anatomy education, which would include applications of modern imaging (point-of-care ultrasound, three-dimensional imaging) tools and simulation technologies that are increasingly relevant in modern anatomy teaching.^6,11^ The focus on education technologies in anatomy is particularly useful given the wide array of modern tools that were developed, optimized, and applied to delivering anatomy education during the pandemic.^12,13^ The second course that was included at this stage (Interprofessional education in the health professions) would emphasize theoretical and evidence-based practical considerations for promoting interprofessional, collaborative competencies in learners. This course will include the study of modern teaching and learning principles, curricular design, and evaluation strategies used as best practices for health profession students in interprofessional contexts. Anatomy laboratory-based education offers a unique environment to promote these valuable skills in future healthcare professionals.^14^

### Enhancing Applied Teaching Opportunities to Include Various learners

As initially described, participants in this program would develop teaching skills and experience by serving as small-group laboratory teachers in the faculty's MD Program. However, external reviewers noted that there would be great benefits to providing experiential teaching opportunities in various settings to various learners. Based on this feedback, efforts were made to provide participants additional teaching opportunities during their second year in the program, including serving as anatomy instructors in other sessions that incorporate lab-based demonstrations, including sessions involving undergraduate, graduate, and high school (eg, during outreach events) learners.

### Ensuring Appropriate Levels of Faculty to Sustain the Program

Another concern brought forward during the external review process was the number of faculty in place to support the program. There is a substantial amount of research expertise in the Faculty of Education and the Faculty of Medicine's Department of Innovation in Medical Education to support ASE graduate students during the progression of their research papers. However, the external review process did highlight a concern with the small number of core faculty dedicated to the anatomical sciences, especially relative to comparable programs. For the program's initial launch, the core faculty in place will drive the delivery of content and the leadership in courses, with support in laboratory teaching environments provided by instructors who have contributed to similar lab-based teaching in the MD Program. As the program grows and evolves during its formative years, any need for more tenure-track professors will be clarified, and such a need will have to be addressed with future recruitment.

The concerns raised during the external review in May 2021 were carefully considered and movement was made to address these concerns from both a faculty and an institutional level, as described above. In the eyes of the same external reviewers, the concerns were deemed to be sufficiently addressed during the summer of 2021, culminating in provincial (Ontario, Canada) approval in December 2021 and successful accreditation from the provincial government to commence in September 2023.^15^

## The Admission Criteria

To be eligible for the program, candidates must have a Bachelor of Science (B.Sc.) degree with a specialization (or equivalent) in biology, biochemistry, pharmacology, physiology, human kinetics (kinesiology), biopharmaceutical, or biomedical or health sciences, with a minimum average of 75% to 79% (B+). Preference will be given to candidates who have completed an undergraduate course in human anatomy (or an equivalent). Candidates who have established a level of anatomy training comparable to the courses offered in the program's first year, as assessed by the program director, can be accepted into a 1-year version of the master's program with a focus on applied teaching and scholarship courses. Candidates are also encouraged to document research or scholarly abilities (via research reports, abstracts, or presentations) in a letter of intent. Applicants must understand and fluently speak the language of instruction (English or French) in the program to which they are applying. Finally, this program will likely attract highly trained international medical graduates (IMGs) to facilitate their career progression in pedagogy. The division currently attracts IMGs in great numbers as teaching assistants in our anatomy laboratories. These IMGs commonly participate in certificate programs in education scholarship (such as the Healthcare Education Scholars Program) and will assuredly be invested in participating in this graduate program.

## Description of Approved, Final Program

After the external review process and adaptations to address reviewer concerns, the governing provincial body approved the final iteration of the program in December 2021.^15^ In its final iteration, the program is described as having 3 goals:

- For students who are aiming for a career in the health profession, this program will provide an excellent foundation in anatomy education that is necessary to treat future patients competently. At the same time, this anatomical expertise will ensure students are highly competitive in pursuing further training (eg, pursuing a degree in medicine, nursing, dentistry, or physiotherapy).
- For students aiming for a career in health profession education and scholarship, this program will provide them with experience, knowledge, and mentorship in education research. Therefore, this program will benefit students aiming for further training (ie, a PhD in health professions education) or a position (ie, contributing to an institutional, national, or private research unit) in a related research field.
- Finally, there is a long-standing and well-established lack of individuals in Canada, the United States, and Europe capable of teaching human anatomy, particularly in a cadaveric laboratory-based setting.^1^ This program is intended to prepare individuals who can excel in delivering modern human anatomy education to various groups of students in a wide range of settings and address the global shortage of competent anatomy educators.

These proposed benefits for learners are supported by the notion that, in the previously described comparative program at Queen's University, the 3 most frequent career path outcomes (within 1 year of graduation) included enrollment in a professional health care degree (medicine, dentistry), the pursuit of studies at the PhD level, and careers in curriculum development and teaching at the postsecondary level.^4^

This 20-month program is comparable in length to other programs offered in Canada and the United States with similar content (ASE), training as an educator [M.Ed.], and training in education scholarship (Master of Arts in Education [MA]). This program is typically offered at the master's level with a thesis at comparable international universities. In Ontario, Queen's University offers a 16-month Master of Science in Anatomical Sciences program, characterized in a 2018 publication.^4^ This program's graduates indicated that they would have benefitted from (i) a greater depth of education scholarship training, (ii) more opportunities to apply education scholarship training in formal dissemination opportunities, and (iii) increased training related to best teaching practices. It is worth noting that many of the other master-level programs in ASE in North America do not have robust levels of education scholarship output.^2^ As such, our proposed program's increased length (relative to the 16-month approach at Queen's University) helps address these issues by giving candidates a complete scholarship experience, including disseminating education scholarship in the required research paper component. Furthermore, a 20-month program would enable candidates to put their anatomy educator training into practice by allowing them to serve as teachers in all parts of the undergraduate medical program (MD program). The experience of teaching all aspects of anatomy in the context of laboratory dissection would be a strength for graduates who intend to apply for anatomy teaching intensive or pathology assistant positions, proven areas of need across North America.

Finally, besides the hands-on learning and skill development gained from anatomy laboratories and cadaveric dissection, the courses offered in this program emphasize student-centered learning approaches based on the Anatomy Division's experience in innovative methods, such as team-based learning,^7^ consistent with modern advances in education in this field.^10^ The active learning principles that form the core of this program are consistent with desired qualifications commonly noted in job descriptions in anatomy education.^1^

The Master in Applied Science in Anatomical Science Education is distinct because it focuses on ASE, including hands-on, applied teaching skills in the context of the human (cadaveric) anatomy laboratory. This training includes human-anatomy dissection, which is vital for teaching anatomy in many allied health professional programs across North America and numerous undergraduate institutions.

The coherence in the Master in Applied Science in Anatomical Science Education can be viewed in a detailed description of the program in Table 2. All courses required are semester-long 12-session, 36-h courses (considered 3 Unit courses locally) that will require a C + minimum grade, except for the ASE7998 Research Paper course and the Seminar in Health Professions Education course, which will both require a grade of Satisfactory. Evaluation of the Research Paper will be modeled on the evaluation approach used by another Master of Science Degree in our Faculty: https://www.uottawa.ca/faculty-medicine/graduate-postdoctoral/students-hub/research-paper-guidelines-epidemiology. Specifically, evaluation of the final scholarly product will be performed by the student's mentor and 1 other Faculty member involved in student supervision in the program and deemed to be Satisfactory if the product meets expectations of scholarly rigor and is judged to be of comparable quality to published articles in the same field. Graduation will require the completion of all 42 Units as described above. Additionally, students will also be encouraged to present their Research Paper at Meridith Marks Day, our local conference dedicated to medical education research and scholarship, to further apply their scholarly dissemination skills: https://www.uottawa.ca/faculty-medicine/department-innovation/educational-events/medical-education-day/meridith-marks-day.

**Table 2.** Compulsory Courses.

CODE	TITLE	UNITS
**Compulsory courses, (42 units):**		
ASE 5101	Anatomy I: Anatomy of the Musculoskeletal System	3 Units
ASE 5102	Anatomy II: Anatomy of the Abdomen: Gastrointestinal, Renal, and Reproductive Systems	3 Units
ASE 5103	Anatomy III: Anatomy of the Head, Neck, and Thorax: Neuroanatomy, Cardiovascular, and Respiratory Systems	3 Units
ASE 5105	Applied Anatomy I	3 Units
ASE 5106	Applied Anatomy II	3 Units
ASE 5107	Histology and Embryology	3 Units
ASE 5966	Séminaire en enseignement aux professionnels de la santé/ Seminar in Health Professions Education	3 Units
ASE 5108	Pathologie humaine/Human Pathology	3 Units
ASE 5909	Formation d’anatomie et d’écho ciblée appliquées /Applied Point-of-Care Ultrasound and Anatomy Bootcamp	3 Units
ASE 7998	Recherche en éducation: Projet de recherche / Education Scholarship: Research Project	6 Units
EDU 5190	Introduction to Research in Education	3 Units
EDU 5105	Interprofessional Education in the Health Professions	3 Units
EDU 5286	Technology and Health Professions Education	3 Units

## Future Directions

To complement standard knowledge-based assessments, observation-based assessment strategies will need to be developed that are appropriate to monitor the progression of teaching competencies and behaviors and to ensure students are graduating with the necessary educational skill sets. Further, the formative years of program evaluation and individual course evaluation will be critical to ensure that teaching and assessment strategies are optimally employed so that students are successfully meeting course-level expectations and program-level learning outcomes. Evidence-based teaching strategies, including best practices and applications of cognitive learning theories and assessment approaches (including spaced repetition, progress testing, and scaffolding) that support the evolving independence of learners as teachers, will need to be continually evaluated and purposefully employed. While this paper only focuses on the program development and approval phase, a subsequent report 5 to 10 years after the program launch can characterize how this ASE program fares during the initial accreditation phase and include data (currently scarcely reported in the literature) regarding career path outcomes for the first several cohorts of graduates. Finally, there was a sudden and dramatic shift in anatomy education forced by the coronavirus disease 2019 (COVID-19) pandemic, which created a massive shift to virtual education in anatomy education and generally limited (at least in the short-term) student opportunities for in-person learning opportunities in the anatomy laboratory. Lessons learned from this era will need to be integrated into forward planning and delivery in this program. These lessons include how to best adapt, optimize and maintain hands-on cadaveric-based education in the context of stringent public health restrictions. It also ensures that students are familiar with and can be flexible in transitioning to best practices in virtual anatomy education approaches when needed.

## Conclusion

In this descriptive article, the authors outline the design of a unique M.Sc. program in anatomical sciences, and the authors hope that this outline is useful for other institutions and individuals who aspire to develop a similar program. The external evaluation of this program suggests that it will be a significantly valuable experience for its graduates in enhancing their anatomy learning and teaching experiences and in subsequent successful employment outcomes.

Authors anticipate that this report will prompt further refinement and improvement in this program and beyond, ultimately providing many more students valuable opportunities to extend and deepen their learning and grow as anatomical science scholars. While this paper only focuses on the program development and approval phase, a subsequent report 5 to 10 years after program launch can characterize how this ASE program fares during the initial accreditation phase and include data (currently scarcely reported in the literature) regarding career path outcomes for the first several cohorts of graduates.

## Supplemental Material

sj-docx-1-mde-10.1177_23821205231183866 - Supplemental material for An Innovative Master in Anatomy: Combining Anatomy With Educational Scholarship Click here for additional data file. Supplemental material, sj-docx-1-mde-10.1177_23821205231183866 for An Innovative Master in Anatomy: Combining Anatomy With Educational Scholarship by Alireza Jalali and Christopher J. Ramnanan in Journal of Medical Education and Curricular Development

sj-docx-2-mde-10.1177_23821205231183866 - Supplemental material for An Innovative Master in Anatomy: Combining Anatomy With Educational Scholarship Click here for additional data file. Supplemental material, sj-docx-2-mde-10.1177_23821205231183866 for An Innovative Master in Anatomy: Combining Anatomy With Educational Scholarship by Alireza Jalali and Christopher J. Ramnanan in Journal of Medical Education and Curricular Development
