# Supplementary material for: An Innovative Master in Anatomy: Combining Anatomy With Educational Scholarship
Source: J Med Educ Curric Dev. 2023 Jun 15;10:23821205231183866. doi: 10.1177/23821205231183866 (PMC10278392; doi:10.1177/23821205231183866)
Supplement: sj-docx-1-mde-10.1177_23821205231183866 - Supplemental material for An Innovative Master in Anatomy: Combining Anatomy With Educational Scholarship [file sj-docx-1-mde-10.1177_23821205231183866.docx]

| **Degree Level Expectations** | **Learning Outcomes** | **How the Program Supports and Evaluates the Outcomes** |
| --- | --- | --- |
| 1. Depth and Breadth of Knowledge | a. Develop anatomy knowledge to a level consistent with modern healthcare professional programs  b. Develop knowledge related to education research and scholarship  c. Develop knowledge related to the application of education technology (including simulation technology) in health professions education  d. Acquire knowledge related to the research, theory, and practice supporting best practices in inter-professional education. | a. Written and practical (lab-based) examinations, formative assessments in cadaver-based anatomical science courses ASE5101/ASE5501, AASE5102/AASE5502, and ASE5103/ASE5503  b. Written examinations in pedagogical and education courses EDU5190/EDU5590, EDU5105/EDU5505, and EDU5286/EDU5686; feedback and evaluation from mentor in ASE7998 |
| 1. Research and Scholarship | a. Develop knowledge related to foundations of education scholarship  b. Can define research questions  c. Can generate research findings of publication quality  c. Demonstrate the appropriate use of research methodologies  d. Interpret results of data gathering using appropriate quantitative or qualitative analysis  e. can critically assess literature in medical education, health professions education, and anatomical sciences education | a. Written examinations in education EDU5190/EDU5590, EDU5105/EDU5505, and EDU5286/EDU5686  b. Progression through research paper, feedback from mentor in ASE7998  c. Mentorship and guidance from faculty; successful presentation and/or publication of work derived from ASE7998; presentations in ASE5366 |
| 1. Level of Application of Knowledge | a. Instruct anatomy in teaching laboratories for MD students  b. Apply pedagogical principles in student-centered teaching environments  c. Apply hands-on dissection skills in anatomy laboratories  d. Evaluate the strengths and limitations of research approaches in relation to their research  e. Apply best practices in the use of modern teaching technologies  f. Apply best practices in the delivery and assessment of inter-professional education | a. Teaching evaluations from MD students and Faculty in applied anatomy courses ASE5105/ASE5505 and ASE5106/ASE5506  b. Teaching evaluations and feedback from MD students and Faculty in applied anatomy courses ANA5105/ANA5505 and ASE5106/ASE5506  c. Feedback from prosector staff and assessment by Faculty in ASE5105/ASE5505 and ASE5106/ASE5506  d. Mentorship, guidance and feedback from Faculty in ASE7998  e. Formative and summative assessments in EDU5190/EDU5590, EDU5105/EDU5505, and EDU5286/EDU5686 |
| 1. Professional Capacity/ Autonomy | a. Display professional attitudes and behaviours to MD students and Faculty in all teaching roles  b. Practice rigour, honesty and integrity in conducting experiments and analyzing findings  c. Successfully demonstrate professionalism in team-based learning environments and teaching environments when working with cadaveric donors from the community  d. Understand the criteria for authorship and the responsibilities therein  e. Understand the ethical principles established by local, provincial and federal agencies that guide the responsible use of human subjects in research  f. Understand the importance of identifying and considering possible sex and gender differences in experimental design  g. Can identify personal, intellectual and financial conflicts of interest  h. Understand confidentiality and unconscious bias in peer review. | a. Teaching evaluations from MD students and Faculty in ASE5105/ASE5505 and ASE5106/ASE5506  b. Feedback from Faculty during ASE7998  c. Feedback from instructors and teaching staff involved in ASE5101/ASE5501, ASE5102/ASE5502, ASE5103/ASE5503, ASE5105/ASE5505, and ASE5106/ASE5506 |
| 1. Level of Communication Skills | a. Develop communication skills relevant to modern medical educators  b. Communicate complex ideas, issues, arguments and research findings clearly and effectively in both oral and written formats in a manner that is accessible and appropriate to a variety of healthcare professionals/learners  c. Can communicate scientific findings to scientific and lay audiences | a. Teaching evaluations from MD students and Faculty in ANA5105/ASE5505 and ASE5106/ASE5506  b. Assessment from Faculty mentor(s) in ASE7998; feedback from peers and Faculty for presentations in ASE5104/ASE5504; participation in seminar course ASE5366  c. Evaluation of written and oral presentations in EDU5190/EDU5590, EDU5105/EDU5505, and EDU5286/EDU5686 |
| 1. Awareness of Limits of Knowledge | a. Perform self-assessment and critical self-reflection regarding development of knowledge  b. Perform self-assessment and critical self-reflection regarding personal development as teacher and scholar | a. Regular formative feedback (quizzes) to monitor development of knowledge in ASE5101/ASE5501, ASE5102/ASE5502, and ASE5103/ASE5503  b. Written self-assessments evaluated by mentors; regular meetings with mentor(s) in ASE5105/ASE5505, ASE5106/ASE5506, and ASE7998 |

Course codes

ASE 5101 Anatomy I: Anatomy of the Musculoskeletal System

ASE 5102 Anatomy II: Anatomy of the Abdomen: Gastrointestinal, Renal and Reproductive Systems

ASE 5103 Anatomy III: Anatomy of the Head, Neck Thorax 3 Units

ASE 5105 Applied Anatomy I

ASE 5106 Applied Anatomy II

ASE 5107 Histology and Embryology

ASE 5908 Human Pathology

ASE 5909 Applied Point-of-Care US and Anatomy Bootcamp

EDU 5105 Inter-Professional Education in the Health Professions

EDU 5190 Introduction to Research in Education

EDU 5286 Technology and Health Professions Education

MED 8166 Professionalism and Professional Skills Seminar

ASE 5966 Seminar in Health Professions Education

Research Project

ASE 7998 Education Scholarship: Research Project 6 Units
