# Supplementary material for: An Innovative Master in Anatomy: Combining Anatomy With Educational Scholarship
Source: J Med Educ Curric Dev. 2023 Jun 15;10:23821205231183866. doi: 10.1177/23821205231183866 (PMC10278392; doi:10.1177/23821205231183866)
Supplement: sj-docx-2-mde-10.1177_23821205231183866 - Supplemental material for An Innovative Master in Anatomy: Combining Anatomy With Educational Scholarship [file sj-docx-2-mde-10.1177_23821205231183866.docx]

# Anatomy Graduate Program Survey

**DEMOGRAPHICS: Please check the appropriate lines:**

**1.** I am currently registered in a Graduate Program in:

______ CMM

______ BMI

______ EPI

**2.** What is your highest level of education?

______ Bachelors Degree

______ Masters Degree

______ Doctorate

______ Other

**Needs Assessment**

**3.** The level of education and training of anatomy should be advanced to

include a graduate degree. (e.g., Master’s of Anatomical Science Education)

**4.** A master’s degree in anatomy would increase the levels of applied anatomical sciences knowledge in future healthcare professionals.

**5.** A master’s degree in anatomical sciences education would increase the levels of clinical

competence of future healthcare professionals.

**6.** The subject area(s) of most need to healthcare professionals is: (rank in order 1=most, 10= least)

- Clinical anatomy
- Histology
- Embryology
- Theoretical anatomy knowledge
- Technical anatomy skills (e.g. Dissection)
- Applied (e.g. Cadaveric-based) teaching skills
- Scholarly dissemination skills
- Research design
- Evidenced-based teaching practice
- Leadership

Additional comments

________________________________________________________________________

________________________________________________________________________

________________________________________________________________________

Thank you completing this survey.
